# A plausible hypothesis for the higher Covid-19 mortality in Brazil

**DOI:** 10.4314/ahs.v23i4.8

**Published:** 2023-12

**Authors:** Heslley Silva

**Affiliations:** University Center of Formiga, Science and Education; University of Minas Gerais State, Education and Science

**Keywords:** Medicines, doctors, Covid-19

## Abstract

Brazil has high Covid-19 mortality rates, especially among those patients who are intubated. It is hypothetically considered that these rates may be related to the abusive use of medicines by the population. These drugs without scientific evidence are indicated by President Bolsonaro and his supporters but are also prescribed by doctors who follow this line. The text draws attention to the risks of this phenomenon.

An investigation by Fiocruz, an important Brazilian research institute, pointed out that in Brazil, the mortality of those who need to be intubated because of Covid-19 is higher than in other countries: 80% of those who need this procedure die in this country, while the mortality in these conditions in other countries is 50% 1. A second stage of this study points out that this difference was increasing in early 2021, the Brazilian picture was worsening. At the same time, the use of unproven drugs is intensifying by indications from the Brazilian government, its supporters, and even doctors 2,3, including the Covid kit (prescribing these drugs together) 4, in a preventive and early approach.

It is clear that the picture that led to this high number of deaths from COVID-19 in Brazil is complex and should not be attributed to just one factor, such as the use of ineffective drugs. It is difficult to judge any action at the beginning of the pandemic period because, at that stage, there was no reasonable protocol for dealing with the disease, so it made some sense to try several drugs that supposedly could have some positive effect 5. However, after 6 months of the pandemic, evidence showed that antimalarial drugs 6, antibiotics 7, anthelmintics 8, among others, were ineffective and possibly dangerous against the SARS-CoV-2 virus^9^. However, a significant portion of the Brazilian medical community, including the Brazilian Federal Council of Medicine, continued to endorse and prescribe these innocuous medications with adverse effects. This sequence of events demonstrates a gap between Brazilian physicians' education (initial and continuing training) and evidence-based practice.

Hypothetically one could relate the intense diffusion of early and preventive treatment to this increase in the severity of Sars-Cov-2 infections. There are obvious possible side effects of that set of drugs, like the risk of patients arriving at hospitals more debilitated by such drugs, ranging from anthelmintics to corticoids, from antimalarials to (believe it or not) antineoplastics. It is also likely a delay in seeking effective help because there would be a false sense of security for those who are undergoing such treatments, making it possible to reach a more advanced stage and difficult recovery.

The simple fact of being able to elaborate on this hypothesis already shows us how risky and absurd it is to adhere to this prescription without any scientific basis 10,11. Maybe we should not attribute the high Brazilian mortality rate exclusively to this factor, but it seems to be an important component of these tragic numbers. This hypothesis would have been dismissed before spreading all this false information through social networks in the world and Brazil about medications against Covid-19 12. However, now it is possible, it is believable because a large part of the Brazilian population is at the mercy and follows these recommendations. Therefore, it is not nonsense to imagine the possibility of this phenomenon interfering with the number of deaths in Brazil.

It is important to highlight that even renowned doctors, locally or regionally, have joined this movement in favor of this kit-covid of early and preventive treatment and have generated pressure so that other doctors, and public administrators, can adhere to this protocol. To corroborate this perception, the municipalities in which Bolsonaro had more votes 13, and consequently more followers who followed him, including on the pharmacological issue, a higher risk of becoming contaminated with Covid-19 and then passing away.

It is possible that there are other factors elevating the risks in Brazil, perhaps the service is precarious, or there is a lack of structure, but this does not seem to be the case because the Brazilian health system is far from being one of the worst in the world. As a matter of fact, the Brazilian public health system (SUS) is recognized for its relevance and its selfless professionals, who face many difficulties, but it continues to be a worldwide highlight as a free health care system 14. The same can be said about Brazilian private hospitals, which are considered efficient in many aspects. Nevertheless, it is important to point out that many physicians on the front line of the pandemic in the reference hospitals in Brazil have reported the complicated picture of several patients who arrive, most of them after having been treated uselessly with this cocktail of various drugs.

An important article illustrates that this hypothesis is not unlikely 15. It shows that the use of the most famous drug in this set, hydroxychloroquine, used since the beginning of the pandemic in Brazil, the one preferred by President Bolsonaro and his followers, including doctors, is associated with increased mortality in patients with Covid-19. The same article has not yet detected any benefit from the use of chloroquine. So, there are only risks, and high ones, no benefits, these Brazilian doctors are prescribing medications based on evidence of their in vitro effectiveness 16, which has not been confirmed in broader research, but its adverse effects have been confirmed 17. The hypothesis advanced here may be turning into a fact, and Brazil is becoming a major testing laboratory to demonstrate this; an indication of this is that the Brazilian Ministry of Health, a year aligned with this type of protocol, no longer recommends the use of the once miraculous chloroquine (May 2021).

It is unwise and unreasonable to credit President Bolsonaro's election alone with the failure of the COVID-19 policy in Brazil 18. Perhaps it is possible to reverse the order of cause and effect in this outcome. In fact, the election of such a controversial and science-averse character 19,20 reveals that there is a critical lack in the Brazilian population regarding scientific education, which reaches the medical community. Evidence of this is that on February 13, 2023, more than three years after the beginning of the pandemic, with all the accumulated knowledge analysed from several scientific methodologies, the president of the Brazilian Federal Council of Medicine published a document questioning and supposedly demonstrating the ineffectiveness of the use of masks to contain the transmission of the SARS-CoV-2 virus. Something so obvious and proven by science and practice 21, makes that statement so absurd 22. Therefore, the election of Bolsonaro, this posture of a significant part of Brazilian doctors, demonstrates the disbelief in science in Brazil disseminated in the population, especially through social networks and Fake News 23, the belief in populist and dogmatic religious leaders and, finally, an educational system 24 that does not promote scientific literacy, critical sense, and inquiry-based teaching.
